# Chronic delusional disorder and Chales Bonnet syndrome: differential diagnosis or comorbidity

**DOI:** 10.1192/j.eurpsy.2022.2038

**Published:** 2022-09-01

**Authors:** M.D.C. Molina Liétor, I. Cuevas Iñiguez

**Affiliations:** 1 Hospital Universitario Príncipe de Asturias, Psiquiatría, Alcalá de Henares, Spain; 2 Hospital Principe de Asturias, Psiquiatría, Alcala de Henares, Spain

**Keywords:** Delusional disorder, Chales Bonnet syndrome, blindness

## Abstract

**Introduction:**

Delusional idea disorders are a group of syndromes whose main or unique characteristic is the presence of consolidated delusional ideas that usually have a chronic character and do not fit into other diagnoses such as schizophrenia, affective disorder or other organic diseases. On the other hand, Charles Bonnet syndrome is an organ hallucinosis in whose context visual hallucinations may appear in patients with a visual deficit. Historically, it has been considered that the presence of another psychiatric condition is an exclusion criterion for the diagnosis of Charles Bonnet syndrome, although the presence of similar etiological and maintenance factors means that this situation of dignous exclusion must be reconsidered.

**Objectives:**

The objective of the present communication is to study the current state of the topics “delusional disorder” and “Charles Bonnet syndrome”. Another objective is to reconsider that the presence of previous or concurrent psychiatric pathology is an exclusion criterion for the diagnosis of Charles Bonnet syndrome..

**Methods:**

A bibliographic review on “delusional ideas disorder” and “Charles Bonnet syndrome” has been carried out, as well as a discussion on the diagnostic and exclusion criteria, based on the etiopathogenic and maintenance factors.

**Results:**

Both in “delusional ideas disorder” and in “charles bonnet syndrome” advanced age, social isolation and deficiencies in sense organs constitute etiological factors that facilitate the appearance of these syndromes and make their treatment difficult.

**Conclusions:**

Due to this, we consider that the appearance of another previous or present psychiatric illness should not be an exclusion criterion, both can appear in the same patient.

**Disclosure:**

No significant relationships.

